# Ethylenation of aldehydes to 3-propanal, propanol and propanoic acid derivatives

**DOI:** 10.1038/s41598-017-01950-7

**Published:** 2017-05-11

**Authors:** Daniel T. Payne, Yiming Zhao, John S. Fossey

**Affiliations:** School of Chemistry, University of Birmingham, Edgbaston, Birmingham, West Midlands B15 2TT UK

## Abstract

Methodology has been developed for the synthesis of 3-propanaldehydes through a five-step process in 11–67% yield from aldehydes. Aldehydes were reacted with Meldrum’s acid through a Knoevenagel condensation to give materials that upon reduction with sodium borohydride and subsequent hydrolysis decarboxylation generated the corresponding 3-propanoic acid derivatives. The -propanoic acid derivatives were reduced to give 3-propanol derivatives, which were readily oxidised to target 3-propanal derivatives.

## Introduction

Aryl-3-propanaldehydes have demonstrated themselves as synthetically useful in the synthesis of natural products^1^, chiral tetrahydroquinolines^2, 3^ chemosensors^4, 5^ and in the perfume industry^6^. As such, facile synthesis of a range of these derivatives would be advantageous.

The chemoselective reduction of cinnamaldehydes to hydrocinnamaldehydes has been reported by Hashizume *et al*. and List *et al. via* either a palladium catalysed reduction^7^ or the organocatalysed Hantzsch’s ester reduction^8^, respectively. The synthesis of cinnamaldehydes has been reported utilising a range of conditions including the Wittig reaction^7^ from aryl aldehydes and the Heck cross-coupling of aryl halides^7, 9–13^. Alternatively, the products from the Knoevenagel condensation of aldehydes with Meldrum’s acid can be converted to hydrocinnamaldehydes. Frost *et al*. reported the hydrosilylation of Meldrum’s acid derivatives (**3**) either through a one-step^14^ or two-step^15^ process, using palladium or molybdenum catalysts and reagents.

A study by Andrews *et al*. (Glaxo-Smith-Kline (GSK)) reported a four-step synthesis of 3-(anthracen-9-yl)propan-1-ol (**6d**) on a 20-gram scale. Upon oxidation, this material would give the corresponding aldehyde (**7d**)^16^. However, this route was reported to have been carried out on a single substrate, starting with 9-anthraldehyde (**1d**) affording 3-(anthracen-9-yl)propan-1-ol (**6d**) in an overall yield of 84% over four steps.

**Figure 1:**
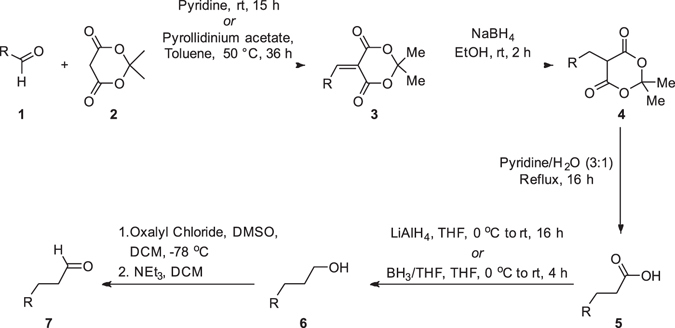
General route for the synthesis of hydrocinnamaldehydes.

Herein we provide alternative methodology to the established literature and build on previous studies^16^ for the synthesis of 3-propanal derivatives (Fig. 1) utilising a Knoevenagel condensation, olefin reduction, decarboxylation, carboxylic acid reduction and an alcohol oxidation. Substrate scope is expanded and a range of versatile hydrocinnamaldehyde derivatives are synthesised.

## Results and Discussion

**Figure 2:**
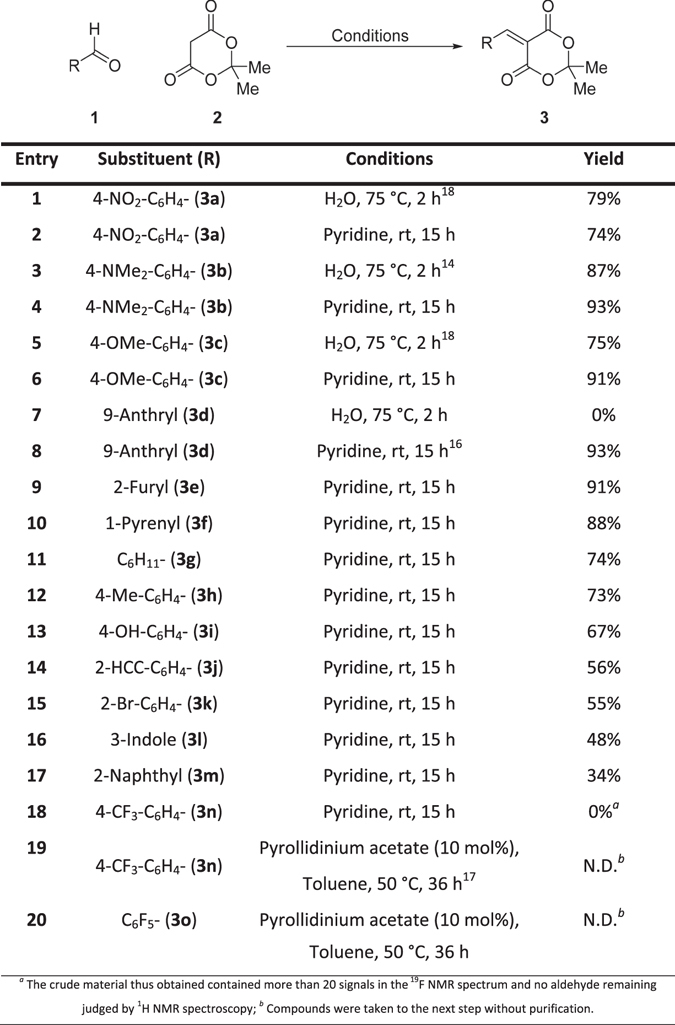
Substrate scope for the Knoevenangel condensation of aldehydes with Meldrum’s acid.

The synthesis of condensation products *para*-nitro (**3a**), para-dimethylamino (**3b**) and para-methoxy (**3c**) could be achieved *via* the literature reported Knoevenagel condensation of aldehydes **1a–c** with Meldrum’s acid (**2**) in 74**–**87% yields^14^. Whilst this method successfully delivered **3a–c** in our hands, the use of an aqueous solvent system prevented us from successfully applying the same conditions to substrates with low water solubility such as 9-anthryl (**3d**, Fig. 2, entry 7). The issue was overcome utilising the method reported by Andrews *et al*. (GSK) for the synthesis of **3d**, where pyridine is used as the reaction solvent^16^. Pleasingly, in contrast to the aqueous solvent system the reaction proceeded smoothly with the 9-anthryl derivative **3d** in 93% yield. We expanded the substrate scope of these conditions to include electron rich (**3b**,**c**,**h**,**I**, Fig. 2, entry 4,6,12,13), electron poor (**3a**, Fig. 2, entry 2), heterocyclic (**3e**,**l**, Fig. 2, entry 9,16), alkyl (**3g**, Fig. 2, entry 11) and hindered (**3f**,**j**,**k**,**m**, Fig. 2, entry 10,14,15,17) groups yielding the desired products in good to moderate yields (34–93%). On the other hand, extremely electron-deficient substrates such as *para*-trifluoromethyl (**3n**, Fig. 2, entry 18) were amenable to this procedure, e.g., decomposition of the starting material was observed.

A literature reported method for the synthesis of **3n** was used^17^, for which we carried out minor solvent modifications to avoid the use of benzene (Fig. 2, entry 19) giving the desired Knoevenagel condensation products. The same procedure also yielded the novel pentafluorophenyl derivative (**3o**, Fig. 2, entry 20), Both the para-trifluoromethyl (**3n**) and pentafluoro (**3o**) derivatives were not purified at this stage due to instability of the substrates during attempted purification protocol, which included recrystallisation and flash column chromatography. Instead, when full conversion was determined to have been reached by ^1^H NMR spectroscopic analysis of the crude reaction mixtures for these reactions, they were taken forward to the next step^18^.

**Figure 3:**
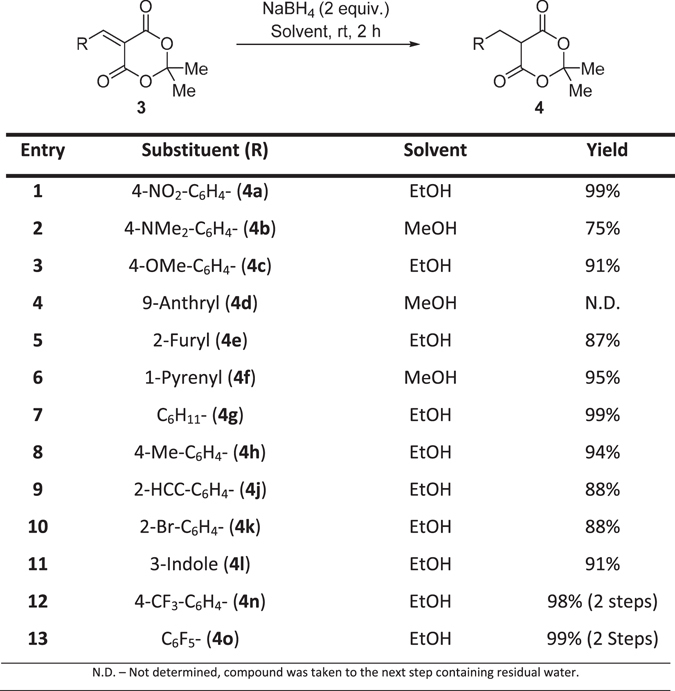
Reduction of Knoevenangel products to afford saturated Meldrum’s derivatives.

With alkene containing compounds **3a–o** in hand, the next step was reduction of the conjugated double bonds introduced through the Knoevenagel condensation. This was successfully carried out according to the method reported for the synthesis of **4d** by Andrews *et al*.^16^ giving high yields (87**–**99%) for **4a**,**c–h**,**j–l**,**n–o**. The 4-dimethylamino derivative (**4b**) gave a lower than expected yield of 75%, minor decomposition was observed. In the case of compounds **4c** (Fig. 3, entry 3) and **4h** (Fig. 3, entry 8) methanol led to ring opening of the Meldrum’s moiety to the dimethyl malonate, whereas under otherwise identical conditions the use of ethanol furnished the desired compounds. Therefore, ethanol was selected as the preferable solvent for manipulation of **3** to **4** from this point.

**Figure 4:**
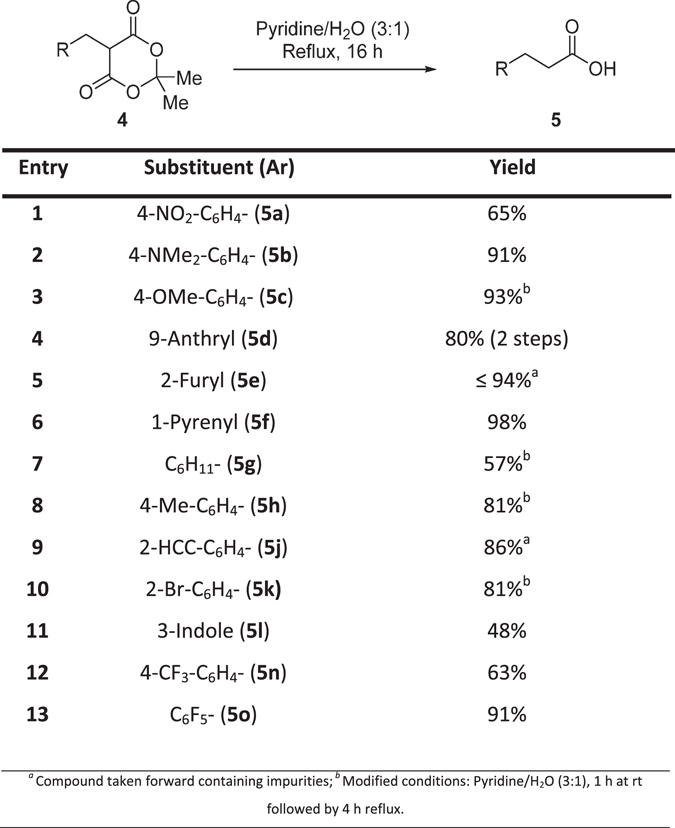
Decarboxylation to synthesise hydrocinnamic acid derivatives.

The hydrolysis and decarboxylation of derivatives **4** was required in order to synthesise **5**, this was achieved with the method reported for the synthesis of **5d** by Andrews *et al*.^16^ in acceptable to good yields (48–98%, Fig. 4) for **5a–h**,**j–l**,**n–o**.

For the synthesis of the *para*-methyl (**5h**) and *para*-methoxy (**5c**) derivatives from **4h** and **4c**, respectively, undesired side-products were detected. In order to minimise the formation of the side-products, the reaction was run initially at room temperature for one hour, followed by heating to reflux for a further 4 hours. The desired compounds were obtained after work-up without requiring further purification. Furthermore, under the standard reaction conditions the synthesis of 2-furyl derivative **5e** from **4e** led to the formation of the desired compound alongside a minor undesired side-product, the desired compound was poorly soluble in common laboratory solvents and therefore this impurity was taken through to the LiAlH_4_ reduction. The low yield for the synthesis of 3-indole derivative **5l** was most likely due to product loss during reaction work-up because of the probable zwitterionic nature of **5l** having some water solubility.

**Figure 5:**
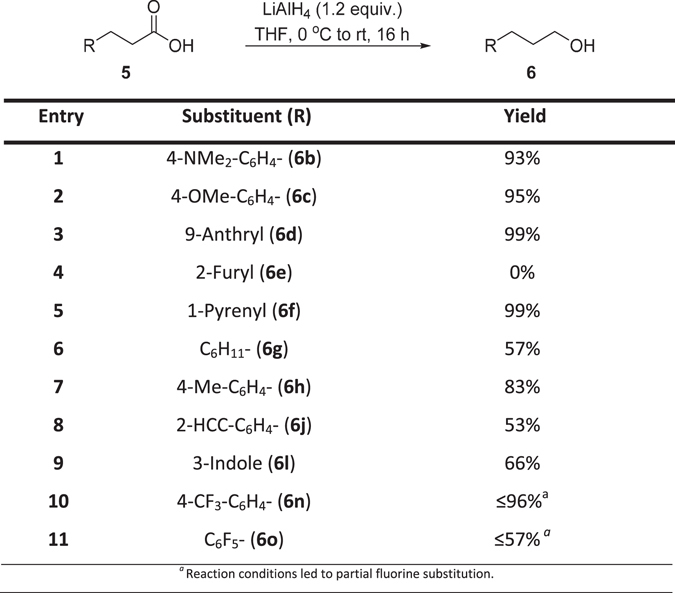
Reduction of carboxylic acids to afford hydrocinnamyl alcohol.

In order to synthesis **7**, isolated **5a–h**,**j–l**,**n–o** should first be converted to the corresponding primary alcohols **6a–h**,**j–l**,**n–o** before oxidation to aldehydes **7a–h**,**j–l**,**n–o**. The reduction of **5b–d, f–h**,**j–l** to **6b–d, f–h**,**j–l** was carried out with lithium aluminium hydride (LiAlH_4_) in THF to give the primary alcohols in 83% to 99% yields (Fig. 5). The reduction of **5e** to **6e** was attempted with lithium aluminium hydride (LiAlH_4_) in THF led to the formation of a number of unidentified decomposition products.

**Figure 6:**
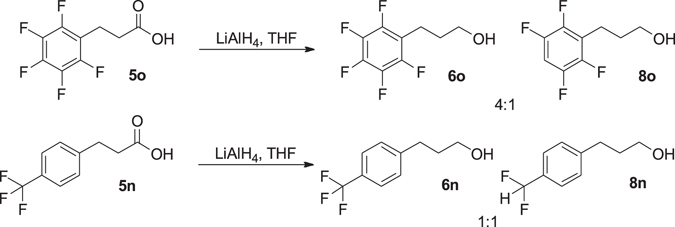
By-products formed during the lithium aluminium hydride reduction of fluorinated hydrocinnamic acids.

The reduction of **5n** and **5o** to **6n** and **6o** was attempted with lithium aluminium hydride (LiAlH_4_) however partial fluorine displacement was observed. Pentafluoro derivative **5o** underwent a nucleophilic aromatic substitution (S_N_Ar) displacing one of the fluorine substituents to give **8o** in an approximate 4:1 ratio **6o**:**8o** (Fig. 6), similar observations are reported in the literature with related substrates^19^. When *para*-trifluoromethyl derivative **6n** was exposed to LiAlH_4_ it underwent a hydride-fluorine exchange to give the *para-*difluoromethyl compound **8n** (Fig. 6) in an approximate 1:1 ratio **6n**:**8n** by ^1^H NMR spectroscopic analysis. Fluorine substitution by hydride within trifluoromethyl groups has been previously reported with related substrates^20^.

**Figure 7:**
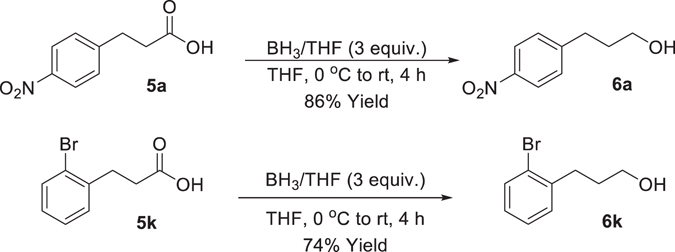
Borane reduction of 4-nitro **5b** and 2-bromo **5k** derivatives.

Reduction of **5a** and **5k** to **6a** and **6k** was carried out using borane to give the desired compounds in 86% and 74%, respectively (Fig. 7). This procedure provides an alternative, milder, method to reduce carboxylic acids when incompatible with LiAlH_4_. Thus, this procedure should also be applicable to fluorinated derivatives **5n** and **5o** and has previously been demonstrated in the literature^21, 22^.

**Figure 8:**
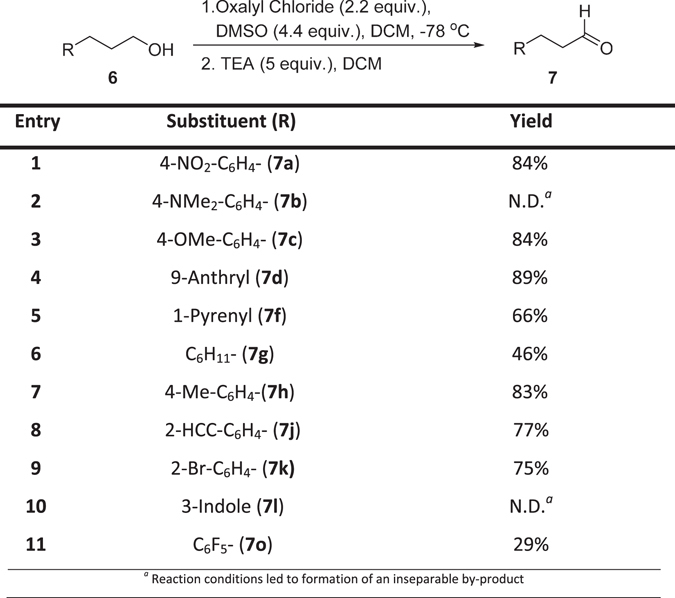
Swern oxidation of cinnamoyl alcohols to give corresponding hydrocinnamaldehyde derivatives.

Hydrocinnamyl alcohol derivatives **6 a,c,d,f–h,j,k,o** were converted to aldehydes **7a,c,d,f–h,j,k,o** using a Swern oxidation in 29**–**89% yield (Fig. 8). The oxidation of 4-dimethylamino derivative **6b** to **7b** and 3-indole derivative **6l** to **7l** was unsuccessful, a complex mixture of unidentifiable by-products alongside the desired compound precluded satisfactory synthesis and isolation. Oxidation of a mixture of **6o** and **8o** led to the formation of the desired aldehyde **7o** in acceptable yield (29%) and the by-product from the oxidation of **8o** could be separated with column chromatography.

**Figure 9:**
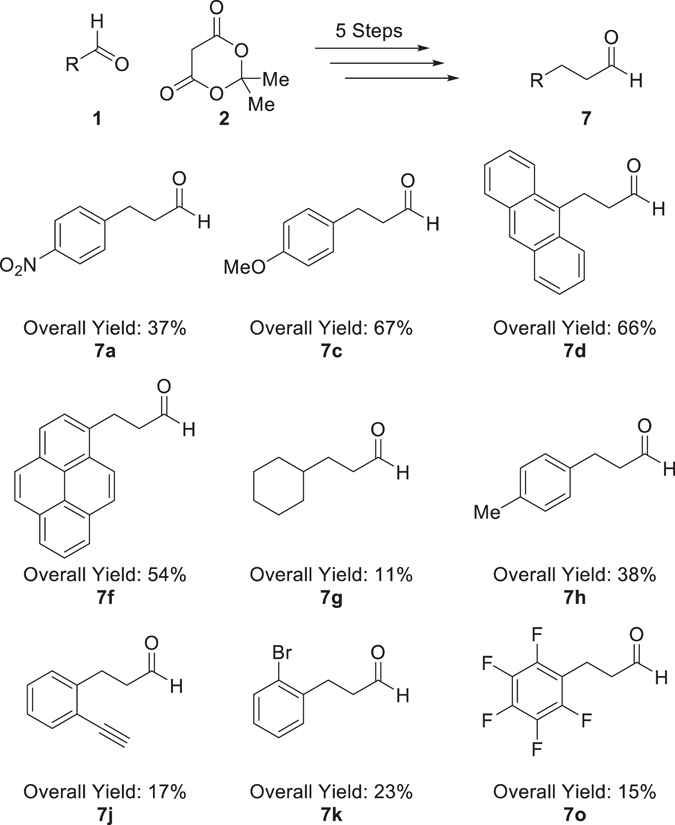
Summary of five step synthesis of hydrocinnamaldehyde derivatives with overall yields.

The outlined five-step synthesis of aldehydes **7** was successful in providing a range of derivatives in acceptable yields (11–67%, Fig. 9). Our studies found that a single set of conditions were not applicable to all substrates but tailoring of reaction conditions can give a diverse range of derivatives. By-products were observed in the LiAlH_4_ reduction of **6n** and **6o**, the decarboxylation of **4d** and **4h** but modifications to the synthetic procedure can minimise their formation^23^. Experimental procedures are detailed in the Supplementary Information.

## Electronic supplementary material

ESI
