# Supplementary material for: Ethylenation of aldehydes to 3-propanal, propanol and propanoic acid derivatives
Source: Sci Rep. 2017 May 11;7:1720. doi: 10.1038/s41598-017-01950-7 (PMC5431831; doi:10.1038/s41598-017-01950-7)
Supplement: Supplementary file 1 — ESI [file 41598_2017_1950_MOESM1_ESM.doc]

# Ethylenation of aldehydes to 3-propanal, propanol and propanoic acid derivatives

Daniel T. Payne, Yiming Zhao and, John S. Fossey

School of Chemistry, University of Birmingham, Edgbaston, Birmingham, West Midlands, B15 2TT, U.K.

[General Experimental 1](#__RefHeading___Toc473201815)

[Experimental Methods and Results 2](#__RefHeading___Toc473201816)

[Knoevenagel Condensation to Synthesise 3a-o 2](#__RefHeading___Toc473201817)

[Knoevenagel General Method 1 2](#__RefHeading___Toc473201818)

[Knoevenagel General Method 2 2](#__RefHeading___Toc473201819)

[Knoevenagel General Method 3 2](#__RefHeading___Toc473201820)

[Olefin Reduction to Synthesise 4a-h,j-l,n,o 8](#__RefHeading___Toc473201821)

[Hydrolysis & Decarboxylation to Synthesise 5a-h,j-l,n,o 14](#__RefHeading___Toc473201822)

[Carboxylic Acid Reduction to Synthesise 6a-h,j,l,n,o 18](#__RefHeading___Toc473201823)

[Carboxylic Acid Reduction General Method 1 18](#__RefHeading___Toc473201824)

[Carboxylic Acid Reduction General Method 2 18](#__RefHeading___Toc473201825)

[Swern Oxidation to Synthesise 7a,c,d,f,g,h,j,k 21](#__RefHeading___Toc473201826)

# General Experimental

Commercially available solvents and reagents were used without further purification. 1H and 19F NMR spectra were recorded on a Bruker AVIII300 NMR spectrometer at 300 MHz and 282 MHz, respectively 1NMR and proton decoupled 13C NMR were recorded at 400 MHz and 101 MHz, respectively, on a Bruker AVIII400 NMR spectrometer at room temperature. All 2D NMR spectra were recorded on a Bruker AVIII400 NMR spectrometer at room temperature. Data was processed on Mestrec version 10.0.2-15465 and Topspin 2.0 (version of Nov 9th 2006). Chemical shifts (δ) are reported in ppm relative to TMS for CDCl3 (δ 0.00) or the residual solvent peak for 1H NMR or relative to the solvent reported for 13C NMR, coupling constants (*J*) are expressed in Hertz (Hz). Shift multiplicities are reported as singlet (s), doublet (d), triplet (t), quartet (q), double doublet (dd), multiplet (m) and broad singlet (bs). Mass spectra were recorded with an electrospray MS Waters LCT time of flight Mass spectrometer or with an EI (GC/MS) Waters GCT Premier Time of Flight Mass Spectrometer. Infrared spectra were recorded on a PerkinElmer 100FT-IR spectrometer at room temperature using an ATR attachment.

# Experimental Methods and Results

## Knoevenagel Condensation to Synthesise 3a-o

### Knoevenagel General Method 1

Aldehyde (1.0 equiv.) and Meldrum’s acid (1.0 equiv.) were suspended in water (0.5 M) and stirred at 75 °C for 2 hrs. The resulting reaction mixture was filtered and the solid residue was washed with water (20 mL/mmol) and petroleum ether (20 mL/mmol) to give the desired compound.

### Knoevenagel General Method 2

Aldehyde (1.0 equiv.) and Meldrum’s acid (1.0 equiv.) were dissolved in Pyridine (0.1 M) and stirred at room temperature overnight under N2. The resulting reaction mixture was reduced to dryness and the resulting solids were either used without further purification or recrystallised.

### Knoevenagel General Method 3

Aldehyde (1.1 equiv.) and Meldrum’s acid (1.0 equiv.) were added to a stirred solution of pyrrolidine (0.1 equiv.) and acetic acid (0.1 equiv.) in anhydrous toluene (0.2M) and stirred at 50 °C for 30 hrs under argon. The solvent was removed under reduced pressure to give the desired compounds which were taken to the next step without purification.

#### 2,2-Dimethyl-5-(4-nitrobenzylidene)-1,3-dioxane-4,6-dione (3a)

Knoevenagel General Method 1: White solid (1.34 g, 79%);

Knoevenagel General Method 2: White solid (2.05 g, 74%);

Mp 219-220 °C; 1H NMR (400 MHz, d6-DMSO) δ 8.51 (s, 1H, C=C*H*), 8.30 (d, *J* = 8.8 Hz, 2H, Ar), 8.07 (d, *J* = 8.8 Hz, 2H, Ar), 1.79 (s, 6H, C*H3*); 13C NMR (101 MHz, d6-DMSO) δ 162.1, 159.5, 154.3, 149.1, 139.0, 132.9, 123.6, 119.6, 105.6, 27.7; IR (neat) ν (cm-1) 1756 (C=O), 1723 (C=O), 1629 (C=C); TOF MS ES+ m/z: 322.0 [M+2Na-H]+, 278.0 [M+H]+.

#### 5-(4-(Dimethylamino)benzylidene)-2,2-dimethyl-1,3-dioxane-4,6-dione (3b)

Knoevenagel General Method 1: Orange solid (2.39 g, 87%);

Knoevenagel General Method 2: Orange solid (5.14 g, 93%);

Mp 169-171 °C (EtOH); 1H NMR (300 MHz, CDCl3) δ 8.30 (s, 1H, C=C*H*), 8.25 (d, *J* = 9.3 Hz, 2H, Ar), 6.69 (d, *J* = 9.3 Hz, 2H, Ar), 3.15 (s, 6H, N(C*H3*)2), 1.77 (s, 6H, C*H3*); 13C NMR (101 MHz, CDCl3) δ 165.2, 161.5, 158.1, 154.5, 139.0, 120.1, 111.2, 105.0, 103.4, 40.1, 27.3; IR (neat) ν (cm-1) 1724 (C=O), 1692 (C=O), 1609 (C=C); TOF MS ES+ m/z: 298.1 [M+Na]+, 232.1 [M- C3H6]+.

#### 5-(4-Methoxybenzylidene)-2,2-dimethyl-1,3-dioxane-4,6-dione (3c)

Knoevenagel General Method 1: Yellow solid (2.73 g, 75%);

Knoevenagel General Method 2: Yellow solid (5.14 g, 91%);

Mp 129-131 °C; 1H NMR (300 MHz, CDCl3) δ 8.38 (s, 1H, C=C*H*), 8.23 (d, *J* = 8.9 Hz, 1H, Ar), 6.99 (d, *J* = 8.9 Hz, 2H, Ar), 3.91 (s, 3H, OC*H3*), 1.79 (s, 6H, C*H3*); 13C NMR (101 MHz, CDCl3) δ 164.7, 164.1, 160.5, 157.9, 137.7, 124.7, 114.4, 110.8, 104.2, 55.7, 27.5; IR (neat) ν (cm-1) 1745 (C=O), 1710 (C=O), 1598 (C=C); TOF MS ES- m/z: 278.3 [M+NH2]-, 176.2 [C10H8O3]-; HR-MS calc. [C14H16O5N]- 278.1034, obs. 278.1027.

#### 5-(Anthracen-9-ylmethylene)-2,2-dimethyl-1,3-dioxane-4,6-dione (3d)

Knoevenagel General Method 2. Orange solid (8.50 g, 93%); Mp 191-192 °C; 1H NMR (300 MHz, CDCl3) δ 9.45 (s, 1H, C=C*H*), 8.53 (s, 1H, Ar), 8.10 – 7.99 (m, 2H, Ar), 7.90 – 7.75 (m, 2H, Ar), 7.60 – 7.40 (m, 4H, Ar), 1.89 (s, 6H, C*H3*); 13C NMR (101 MHz, CDCl3) δ 161.9, 157.9, 157.7, 130.9, 130.3, 129.3, 128.6, 127.1, 126.8, 125.6, 124.5, 121.0, 105.0, 28.2; IR (neat) ν (cm-1) 1766 (C=O), 1731 (C=O), 1621 (C=C); TOF MS ES+ m/z: 355.1 [M+Na]+, 329.1 [M+Li]+, 289.1 [M-C3H6]+; HR-MS calc. [C21H16O4Na]+ 355.0941, obs. 355.0937.

#### 5-(Furan-2-ylmethylene)-2,2-dimethyl-1,3-dioxane-4,6-dione (3e)

Knoevenagel General Method 2. Black solid (1.12 g, 91%); 1H NMR (400 MHz, CDCl3) δ 8.47 (d, *J* = 3.9 Hz, 1H,OC*H=*CH), 8.36 (s, 1H, C=C*H*), 7.86 (dd, *J* = 1.7, 0.7 Hz, 1H, OCC*H*), 6.76 (ddd, *J* = 3.9, 1.7, 0.7 Hz, 1H. OCHC*H*), 1.78 (s, 6H, C*H3*); 13C NMR (101 MHz, CDCl3) δ 163.2, 160.2, 150.4, 150.2, 141.2, 128.1, 115.3, 107.5, 104.5, 27.6; IR (neat) ν (cm-1) 1742 (C=O), 1706 (C=O), 1584 (C=C); TOF MS ES- m/z: 238.3 [M+NH2]-; HR-MS calc. [C11H12O5N]- 238.0721, obs. 238.0712.

#### 2,2-Dimethyl-5-(pyren-1-ylmethylene)-1,3-dioxane-4,6-dione (3f)

Knoevenagel General Method 2. Orange solid (3.16 g, 88%); Mp 205-206 °C; 1H NMR (300 MHz, CDCl3) δ 9.54 (s, 1H, C=C*H*), 8.50 (d, *J* = 7.6 Hz, 1H, Ar), 8.35 – 8.03 (m, 8H, Ar), 1.92 (s, 6H, C*H3*); 13C NMR (101 MHz, CDCl3) δ 163.2, 160.0, 156.3, 135.0, 131.8, 131.0, 130.4, 130.3, 129.9, 128.8, 127.3, 127.1, 127.0, 126.6, 125.6, 124.5, 124.2, 122.7, 115.2, 104.7, 27.9; IR (neat) ν (cm-1) 1763 (C=O), 1731 (C=O), 1603 (C=C), 1592 (C=C); TOF MS ES+ m/z: 395.3 [M+K]+, 379.3 [M+Na]+.

*One carbon attributed to the pyrene was not visible in the 13C NMR spectrum.*

#### 5-(cyclohexylmethylene)-2,2-dimethyl-1,3-dioxane-4,6-dione (3g)

Knoevenagel Gerneral Method 2. White solid (3.53g, 74%); Rf = 0.8 (Hexane/EtOAc = 1:1); Mp 78-80 °C; 1H NMR (300 MHz, CDCl3) δ 7.71 (d, *J* = 10.5 Hz, 1H, C*H*), 3.52 (qt, *J* = 10.0, 2.9 Hz, 1H, (CH2)2C*H*CH), 1.74 (m, 10H, Cy & C*H3*), 1.51 – 0.88 (m, 6H, Cy); 13C NMR (101 MHz, CDCl3) δ 172.2, 162.3, 159.7, 116.3, 104.8, 39.2, 31.1, 27.6, 25.5, 24.8; IR(neat) v (cm-1) 1725 (C=O), 1621 (C=O); TOF MS ES- m/z: 254.3 [M-H+NH3]-; HR-MS calc. [C13H20NO4]- 254.1398, obs. 254.1389.

#### 2,2-Dimethyl-5-(4-methylbenzylidene)-1,3-dioxane-4,6-dione (3h)

Knoevenagel General Method 2. White solid (0.32 g, 73%); Mp 126-128 °C; 1H NMR (300 MHz, CDCl3) δ 8.40 (s, 1H, C=C*H*), 8.01 (d, *J* = 8.2 Hz, 2H, Ar), 7.30 (d, *J* = 8.2 Hz, 2H, Ar), 2.44 (s, 3H, PhC*H3*), 1.80 (s, 6H, C*H3*); 13C NMR (101 MHz, CDCl3) δ 163.6, 160.0, 158.3, 145.5, 134.2, 129.6, 129.1, 113.4, 104.4, 27.6, 22.0; IR (neat) ν (cm-1) 1764 (C=O), 1726 (C=O), 1601 (C=C); TOF MS ES- m/z: 262.3 [M+NH2]-, 160.2 [C10H8O2]-; HR-MS calc. [C14H16O4N]- 262.1085, obs. 262.1082.

#### 5-(4-Hydroxybenzylidene)-2,2-dimethyl-1,3-dioxane-4,6-dione (3i)

Knoevenagel General Method 2. Pale yellow solid (0.58 g, 67%); Mp 204-209 °C; 1H NMR (400 MHz, d3-MeOD) δ 8.35 (s, 1H, C=C*H*), 8.20 (d, *J* = 8.9 Hz, 2H, Ar), 6.90 (d, *J* = 8.9 Hz, 2H, Ar), 1.78 (s, 6H, C*H3*); 13C NMR (101 MHz, d3-MeOD) δ 164.4, 164.0, 160.8, 157.8, 137.9, 123.6, 115.4, 109.6, 104.0, 26.0; IR (neat) ν (cm-1) 3276 (OH), 1745 (C=O), 1692 (C=O), 1615 (C=C); TOF MS ES- m/z: 247.3 [M-H]-; HR-MS calc. [C13H11O5]- 247.0612, obs. 247.0604.

#### 5-(2-Ethynylbenzylidene)-2,2-dimethyl-1,3-dioxane-4,6-dione (3j)

Knoevenagel General Method 2. Orange solid (0.50 g, 56%); Mp 136-137 °C; 1H NMR (400 MHz, CDCl3) δ 8.86 (s, 1H, C=C*H*), 7.99 (dd, *J* = 7.9, 1.4 Hz, 1H, Ar), 7.61 (dd, *J* = 7.4, 1.4 Hz, 1H, Ar), 7.47 (ddd, *J* = 7.9, 7.4, 1.4 Hz, 1H, Ar), 7.43 (ddd, *J* = 7.9, 7.4, 1.4 Hz, 1H, Ar), 3.50 (s, 1H C≡C*H*), 1.83 (s, 6H, C*H3*); 13C NMR (101 MHz, CDCl3) δ 162.5, 159.3, 156.0, 134.2, 133.2, 132.0, 130.8, 128.6, 124.6, 117.1, 104.8, 85.1, 80.9, 27.8; IR (neat) ν (cm-1) 3260 (C≡C), 1757 (C=O), 1724 (C=O), 1607 (C=C); TOF MS ES- m/z: 272.3 [M+NH2]-; HR-MS calc. [C15H14O4N]- 272.0928, obs. 272.0921.

#### 5-(2-Bromobenzylidene)-2,2-dimethyl-1,3-dioxane-4,6-dione (3k)

Knoevenagel General Method 2. White solid (0.59 g, 55%); Mp 139-142 °C; 1H NMR (400 MHz, CDCl3) δ 8.61 (s, 1H, C=C*H*), 7.75 (dd, *J* = 7.8, 2.1 Hz, 1H, Ar), 7.67 (dd, *J* = 7.8, 1.6 Hz, 1H, Ar), 7.39 (ddd, *J* = 7.9, 7.6, 1.6 Hz, 1H, Ar), 7.34 (ddd, *J* = 7.9, 7.6, 2.1 Hz, 1H, Ar), 1.83 (s, 6H, C*H3*); 13C NMR (101 MHz, CDCl3) δ 162.1, 158.9, 156.6, 132.9, 132.8, 131.7, 127.0, 125.0, 117.8, 105.0, 27.8; IR (neat) ν (cm-1) 1763 (C=O), 1729 (C=O), 1619 (C=C); TOF MS ES- m/z: 326.3/328.3 [M+NH2]-, 224.2/226.2 [C9H5O2Br]-; HR-MS calc. [C13H13O4NBr79]- 326.0033, obs. 326.0029.

*One carbon attributed to the aromatic ring was not visible in the 13C NMR spectrum.*

#### 5-((1H-Indol-3-yl)methylene)-2,2-dimethyl-1,3-dioxane-4,6-dione (3l)

Knoevenagel General Method 2. Yellow solid (0.45 g, 48%); Mp 254-257 °C (Decomposition at 240 ˚C); 1H NMR (400 MHz, d6-DMSO) δ 12.90 (s, 1H, N*H*), 9.33 (s, 1H, C=C*H*), 8.74 (s, 1H, NHC*H*), 8.06 – 7.84 (m, 1H, Ar), 7.75 – 7.54 (m, 1H, Ar), 7.45 – 7.22 (m, 2H, Ar), 1.71 (s, 6H, C*H3*); 13C NMR (101 MHz, d6-DMSO) δ 164.4, 161.9, 146.6, 140.5, 136.9, 129.3, 124.4, 123.4, 118.4, 113.8, 111.7, 103.9, 103.5, 27.2; IR (neat) ν (cm-1) 3160 (NH), 1730 (C=O), 1675 (C=O), 1600 (C=C); TOF MS ES- m/z: 270.3 [M-H]-; HR-MS calc. [C15H12O4N]- 270.0772, obs. 270.0765.

#### 2,2-Dimethyl-5-(naphthalen-2-ylmethylene)-1,3-dioxane-4,6-dione (3m)

Knoevenagel General Method 2. White solid (0.11 g, 34%); Mp 151-153 ˚C; 1H NMR (300 MHz, CDCl3) δ 8.59 (s, 1H, C=C*H*), 8.56 (s, 1H, Ar), 8.13 (dd, *J* = 8.8, 1.5 Hz, 1H, Ar), 7.95 (d, *J* = 8.1 Hz, 1H, Ar), 7.88 (d, *J* = 8.8 Hz, 1H, Ar), 7.87 (d, *J* = 8.1 Hz, 1H, Ar), 7.64 (ddd, *J* = 8.1, 6.9, 1.5 Hz, 1H, Ar), 7.56 (ddd, *J* = 8.1, 6.9, 1.5 Hz, 1H, Ar), 1.84 (s, 6H, C*H3*); 13C NMR (101 MHz, CDCl3) δ 163.4, 160.0, 158.2, 137.0, 135.7, 132.7, 129.8, 129.4, 129.4, 128.3, 128.1, 127.8, 127.0, 114.4, 104.6, 27.7; IR (neat) ν (cm-1) 1758 (C=O), 1722 (C=O), 1593 (C=C); TOF MS ES- m/z: 298.4 [M+NH2]-, 196.2 [C13H8O2]-; HR-MS calc. [C17H16O4N]- 298.1085, obs. 298.1082.

#### 2,2-Dimethyl-5-(4-(trifluoromethyl)benzylidene)-1,3-dioxane-4,6-dione (3n)

Knoevenagel General Method 3; Cream solid; Mp 126-129 °C; 1H NMR (300 MHz, CDCl3) δ 8.44 (s, 1H, C=C*H*), 8.06 (d, *J* = 8.3 Hz, 2H, Ar), 7.73 (d, *J* = 8.3 Hz, 2H, Ar), 1.83 (s, 6H, C*H3*); 19F NMR (282 MHz, CDCl3) δ -63.32; 13C NMR (101 MHz, CDCl3) δ 162.5, 159.2, 155.8, 134.8, 132.9, 129.0, 126.2, 125.5, 125.5, 105.0, 27.8; TOF MS ES- m/z: 316.4 [M+NH2]-, 214.2 [C10H5O2F3]-; HR-MS calc. [C14H13O4NF3]- 316.0802, obs. 316.0798.

#### 2,2-Dimethyl-5-((perfluorophenyl)methylene)-1,3-dioxane-4,6-dione (3o)

Knoevenagel General Method 3. 1H NMR (300 MHz, CDCl3) δ 8.16 (q, *J* = 1.5 Hz, 1H, C=C*H*), 1.85 (s, 6H, C*H3*); 19F NMR (282 MHz, CDCl3) δ -134.92, -148.31, -161.07; 13C NMR (101 MHz, CDCl3) δ 160.3, 158.5, 143.97 (m), 142.88 (m), 138.9, 137.18 (m), 123.0, 108.63 (td, *J* = 16.3, 3.8 Hz), 105.7, 27.6.

## Olefin Reduction to Synthesise 4a-h,j-l,n,o

Sodium borohydride (2.0 equiv.) was added portionwise over 2 hrs a stirred solution of the Knoevenagel products (**4**, 1.0 equiv.) suspended in either methanol or ethanol (0.1 M). Upon completion, determined by TLC, the reaction was quenched with 1M aqueous HCl (15 mL/mmol) and the precipitate was filtered, washed with water and dried at the pump to afford the desired compound without further purification.

Table 1 - Summary of solvents and yields for the synthesis of **76b-h,j-l,n,o**

| Entry | **Substituent (R)** | **Solvent** | **Yield** |
| --- | --- | --- | --- |
| 1 | 4-NO2-C6H4- (**4a**) | EtOH | 99% |
| 2 | 4-NMe2-C6H4- (**4b**) | MeOH | 75% |
| 3 | 4-OMe-C6H4- (**4c**) | EtOH | 91% |
| 4 | 9-Anthryl (**4d**) | MeOH | N.D.* |
| 5 | 2-Furyl (**4e**) | EtOH | 87% |
| 6 | 1-Pyrenyl (**4f**) | MeOH | 95% |
| 7 | C6H11- (**4g**) | EtOH | 99% |
| 8 | 4-Me-C6H4- (**4h**) | EtOH | 94% |
| 9 | 2-HCC-C6H4- (**4j**) | EtOH | 88% |
| 10 | 2-Br-C6H4- (**4k**) | EtOH | 88% |
| 11 | 3-Indole (**4l**) | EtOH | 91% |
| 12 | 4-CF3-C6H4- (**4n**) | EtOH | 98% (2 steps) |
| 13 | C6F5- (**4o**) | EtOH | 99% (2 Steps) |
| ***Compound was taken to the next step containing residual water** | | | |

#### 2,2-dimethyl-5-(4-nitrobenzyl)-1,3-dioxane-4,6-dione (4a)

White solid (1.52 g, 99%); Mp 142-144 °C; 1H NMR (400 MHz, d6-DMSO) δ 8.15 (d, *J* = 8.5 Hz, 2H, Ar), 7.59 (d, *J* = 8.5 Hz, 2H, Ar), 4.91 (t, *J* = 5.5 Hz, 1H, C*H*CH2), 3.40 (d, *J* = 5.5 Hz, 2H, C*H2*), 1.85 (s, 3H, C*H3*), 1.66 (s, 3H, C*H3*); 13C NMR (101 MHz, CDCl3) δ 170.4, 165.8, 147.3, 146.6, 130.9, 123.6, 105.6, 47.0, 31.4, 28.4, 26.3; IR (neat) ν (cm-1) 1776 (C=O), 1730 (C=O); TOF MS ES- m/z: 278.3 [M-H]-; HR-MS calc. [C13H12O6N]- 278.0670, obs. 278.0667.

#### 5-(4-(Dimethylamino)benzyl)-2,2-dimethyl-1,3-dioxane-4,6-dione (4b)

yellow solid (2.39 g, 75%); Mp 110-112 °C; 1H NMR (300 MHz, CDCl3) δ 7.18 (d, *J* = 8.8 Hz, 2H, Ar), 6.65 (d, *J* = 8.8 Hz, 2H, Ar), 3.71 (t, *J* = 4.8 Hz, 1H, C*H*CH2), 3.41 (d, *J* = 4.8 Hz, 2H, C*H2*), 2.91 (s, 6H, N(C*H3*)2), 1.71 (s, 3H, C*H3*), 1.45 (s, 3H, C*H3*); 13C NMR (101 MHz, CDCl3) δ 165.7, 149.8, 130.6, 124.8, 112.7, 105.2, 48.4, 40.6, 31.6, 28.5, 27.4; IR (neat) ν (cm-1) 1791 (C=O), 1749 (C=O), 1612 (C=C); TOF MS ES+ m/z: 278.3 [M+H]+, 220.2 [M-OC3H6]+.

#### 5-(4-Methoxybenzyl)-2,2-dimethyl-1,3-dioxane-4,6-dione (4c)

White solid (4.80 g, 91%); Rf = 0.56 (Hexane/EtOAc = 3:4); Mp 83-85 °C; 1H NMR (300 MHz, CDCl3) δ 7.24 (d, *J* = 8.7 Hz, 2H, Ar), 6.82 (d, *J* = 8.7 Hz, 2H, Ar), 3.77 (s, 3H, OCH3), 3.73 (t, *J* = 4.9 Hz, 1H, CHCH2), 3.44 (d, *J* = 4.9 Hz, 2H, CH2), 1.72 (s, 3H, CH3), 1.48 (s, 3H, CH3); 13C NMR (101 MHz, CDCl3) δ 165.4, 158.7, 131.0, 129.0, 113.9, 105.2, 55.2, 48.3, 31.5, 28.5, 27.4; IR (neat) ν (cm-1) 1785 (C=O), 1744 (C=O); TOF MS ES+ m/z: 287.2 [M+Na]+; HR-MS calc. [C14H16O5Na]+ 287.0890, obs. 287.0891.

#### 5-(Anthracen-9-ylmethyl)-2,2-dimethyl-1,3-dioxane-4,6-dione (4d)

Yellow solid; 1H NMR (300 MHz, CDCl3) δ 8.58 – 8.35 (m, 3H, Ar), 8.12 – 7.94 (m, 2H, Ar), 7.63 – 7.38 (m, 4H, Ar), 4.47 (d, *J* = 5.2 Hz, 2H, C*H2*), 3.95 (t, *J* = 5.2 Hz, 1H, C*H*CH2), 1.75 (s, 3H, C*H*3), 1.61 (s, 3H, C*H3*); 13C NMR (101 MHz, CDCl3) δ 165.3, 131.6, 130.5, 130.0, 129.4, 127.5, 126.3, 125.0, 124.3, 105.1, 49.1, 28.8, 26.3, 23.9.

#### 5-(Furan-2-ylmethyl)-2,2-dimethyl-1,3-dioxane-4,6-dione (4e)

Black solid (1.12 g, 87%); 1H NMR (300 MHz, CDCl3) δ 7.30 – 7.29 (m, 1H, *H*C=CH), 6.29 (dd, *J* = 3.2, 1.9 Hz, 1H, *H*C=CH), 6.16 (dd, *J* = 3.2, 0.9 Hz, 1H, *H*C=CH), 3.89 (t, *J* = 5.1 Hz, 1H, C*H*CH2), 3.50 (d, *J* = 5.1 Hz, 2H, C*H2*), 1.79 (s, 3H, C*H3*), 1.67 (s, 3H, C*H3*); 13C NMR (101 MHz, CDCl3) δ 164.9, 150.6, 141.5, 110.6, 107.7, 105.2, 45.4, 28.4, 26.9, 24.9; TOF MS ES- m/z: 223.2 [M-H]-; HR-MS calc. [C11H11O5]- 223.0612, obs. 223.0608.

#### 2,2-Dimethyl-5-(pyren-1-ylmethyl)-1,3-dioxane-4,6-dione (4f)

Cream solid (3.16 g, 95%); Mp 171-173 °C; 1H NMR (300 MHz, CDCl3) δ 8.33 (d, *J* = 9.3 Hz, 1H, Ar), 8.23 – 8.12 (m, 5H, Ar), 8.09 – 7.97 (d, *J* = 11.4 Hz, 3H, Ar), 4.21 (d, *J* = 5.1 Hz, 2H, C*H2*), 3.91 (t, *J* = 5.1 Hz, 1H, C*H*CH2), 1.67 (s, 6H, C*H3*); 13C NMR (101 MHz, CDCl3) δ 165.4, 132.2, 131.3, 130.7, 128.9, 128.5, 128.4, 127.5, 127.2, 126.0, 125.4, 125.2, 125.1, 124.9, 124.8, 122.3, 105.2, 94.8, 48.8, 29.3, 28.6, 26.5; IR (neat) ν (cm-1) 1790 (C=O) 1738 (C=O) 1612 (C=C) 1592 (C=C); TOF MS ES- m/z: 357.4 [M-H]-, 255.3 [M-C4H7O3]-; HR-MS calc. [C23H17O4]- 357.1132, obs. 357.1125.

#### 5-(cyclohexylmethyl)-2,2-dimethyl-1,3-dioxane-4,6-dione (4g)

White solid (1.20g, 99%); Mp 98 °C; 1H NMR (300 MHz, CDCl3) δ 3.50 (t, *J* = 5.7 Hz, 1H, (CH2)2CHC*H*), 1.97 (t, *J* = 6.4 Hz, 2H, CHC*H2*CH), 1.80 (s, 3H, C*H3*) 1.75 – 1.60 (m, 9H, Cy & C*H3*), 1.36 – 1.06 (m, 3H, Cy), 1.06 – 0.86 (m, 2H, Cy). 13C NMR (101 MHz, CDCl3) δ 166.1, 104.9, 43.6, 35.2, 33.9, 32.9, 28.6, 26.8, 26.3, 26.0; IR(neat) v (cm-1) 1792 (C=O), 1742 (C=O); TOF MS ES- m/z: 239.3 [M-H]-; HR-MS calc. [C13H19O4]- 239.1278, obs. 239.1284.

#### 2,2-Dimethyl-5-(4-methylbenzyl)-1,3-dioxane-4,6-dione (4h)

White solid (3.12 g, 94%); Rf = 0.53 (Hexane/EtOAc = 1:1); Mp 170-171 °C; 1H NMR (300 MHz, CDCl3) δ 7.20 (d, *J* = 7.7 Hz, 2H, Ar), 7.09 (d, *J* = 7.7 Hz, 2H, Ar), 3.74 (t, *J* = 4.9 Hz, 1H, C*H*), 3.45 (d, *J* = 4.9 Hz, 2H, C*H2*), 2.30 (s, 3H, PhC*H3*), 1.73 (s, 3H, C*H3*), 1.50 (s, 3H, C*H3*); 13C NMR (101 MHz, CDCl3) δ 165.4, 136.8, 134.1, 129.6, 129.3, 105.2, 48.3, 31.8, 28.5, 27.3, 21.0; IR (neat) ν (cm-1) 1785 (C=O), 1745 (C=O); TOF MS AP- m/z: 247.1 [M-H]-; HR-MS calc. [C14H15O4]- 247.0976, obs. 247.0968.

#### 5-(2-Ethynylbenzyl)-2,2-dimethyl-1,3-dioxane-4,6-dione (4j)

Orange solid (0.50 g, 88%); Mp 119-121 °C; 1H NMR (400 MHz, CDCl3) δ 7.51 (dd, *J* = 2.9, 1.4 Hz, 1H, Ar), 7.49 (dd, *J* = 2.9, 1.4 Hz, 1H, Ar), 7.34 (td, *J* = 7.6, 1.4 Hz, 1H, Ar), 7.24 (td, *J* = 7.6, 1.4 Hz, 1H, Ar), 4.21 (t, *J* = 6.0 Hz, 1H, C*H*CH2), 3.55 (d, *J* = 6.0 Hz, 2H, C*H2*), 3.38 (s, 1H, C≡C*H*), 1.80 (s, 3H, C*H3*), 1.76 (s, 3H, C*H3*); 13C NMR (101 MHz, CDCl3) δ 164.8, 140.7, 133.0, 131.2, 129.1, 127.0, 121.1, 105.0, 82.8, 81.6, 47.3, 30.6, 28.7, 26.1; IR (neat) ν (cm-1) 3280 (CH), 1782 (C=O), 1738 (C=O); TOF MS ES- m/z: 257.3 [M-H]-; HR-MS calc. [C15H13O4]- 257.0819, obs. 257.0809.

#### 5-(2-Bromobenzyl)-2,2-dimethyl-1,3-dioxane-4,6-dione (4k)

White solid (0.31 g, 88%); 1H NMR (300 MHz, CDCl3) δ 7.57 (dd, *J* = 3.5, 1.5 Hz, 1H, Ar), 7.54 (dd, *J* = 3.5, 1.5 Hz, 1H, Ar), 7.29 (td, *J* = 7.7, 1.5 Hz, 1H, Ar), 7.14 (td, *J* = 7.7, 1.5 Hz, 1H, Ar), 4.06 (t, *J* = 5.9 Hz, 1H, C*H*CH2), 3.50 (d, *J* = 5.9 Hz, 2H, C*H2*), 1.82 (s, 3H, C*H3*), 1.77 (s, 3H, C*H3*); 13C NMR (101 MHz, CDCl3) δ 164.6, 137.0, 133.2, 132.8, 128.8, 127.5, 124.1, 105.2, 46.7, 32.1, 28.7, 26.2; IR (neat) ν (cm-1) 1791 (C=O), 1744 (C=O); TOF MS ES- m/z: 311.2/313.2 [M-H]-, 209.1/211.1 [M-C4H6O3]-; HR-MS calc. [C13H12O4Br79]- 310.9924, obs. 310.9916.

#### 5-((1H-Indol-3-yl)methyl)-2,2-dimethyl-1,3-dioxane-4,6-dione (4l)

White solid (0.45 g, 91%); Mp 115-117 °C; 1H NMR (400 MHz, CDCl3) δ 8.07 (s, 1H, N*H*), 7.73 (d, *J* = 7.8 Hz, 1H, Ar), 7.34 (d, *J* = 7.8 Hz, 1H, Ar), 7.23 – 7.11 (m, 3H, Ar), 3.78 (t, *J* = 4.7 Hz, 1H, C*H*CH2), 3.67 (d, *J* = 4.7 Hz, 2H, C*H*2), 1.70 (s, 3H, C*H3*), 1.46 (s, 3H, C*H3*); 13C NMR (101 MHz, CDCl3) δ 165.9, 135.8, 127.1, 124.3, 122.2, 119.8, 119.1, 111.5, 111.0, 105.1, 47.8, 28.4, 27.3, 22.2; IR (neat) ν (cm-1) 3419 (NH), 1786 (C=O), 1741 (C=O); TOF MS ES- m/z: 272.3 [M-H]-; HR-MS calc. [C15H14O4N]- 272.0928, obs. 272.0920.

#### 2,2-Dimethyl-5-(4-(trifluoromethyl)benzyl)-1,3-dioxane-4,6-dione (4n)

White solid (0.85 g, 98%); Mp 147-149 °C;  1H NMR (300 MHz, CDCl3) δ 7.56 (d, *J* = 8.2 Hz, 2H, Ar), 7.47 (d, *J* = 8.2 Hz, 2H, Ar), 3.79 (t, *J* = 5.0 Hz, 1H, C*H*CH2), 3.54 (d, *J* = 5.0 Hz, 2H, C*H2*), 1.76 (s, 3H, C*H3*), 1.63 (s, 3H, C*H3*); 19F NMR (282 MHz, CDCl3) δ -62.58; 13C NMR (101 MHz, CDCl3) δ 164.9, 141.2, 130.2, 125.5, 125.5, 105.3, 47.9, 31.5, 28.4, 27.0; IR (neat) ν (cm-1) 1767 (C=O), 1729 (C=O), 1630 (C=C); TOF MS ES- m/z: 301.3 [M-H]-, 199.2 [M-C4H7O3]-; HR-MS calc. [C14H12O4F3]- 301.0693, obs. 301.0689.

*One carbon attributed to the CF3 was not visible in the 13C NMR spectrum.*

#### 2,2-Dimethyl-5-((perfluorophenyl)methyl)-1,3-dioxane-4,6-dione (4o)

White solid (1.65 g, >99%); Mp 120-123 °C; 1H NMR (300 MHz, CDCl3) δ 3.92 (t, *J* = 6.9 Hz, 1H, C*H*CH2), 3.45 (dq, *J* = 6.9, 1.2 Hz, 2H, C*H2*), 1.84 (s, 3H, C*H3*), 1.80 (s, 3H, C*H3*); 19F NMR (282 MHz, CDCl3) δ -142.08 (m), -155.83 (t, *J* = 20.8 Hz), -162.21 (m); 13C NMR (101 MHz, CDCl3) δ 164.0, 105.6, 28.5, 26.5, 19.9; IR (neat) ν (cm-1) 1802 (C=C), 1790 (C=O), 1747 (C=O); TOF MS ES+ m/z: 347.2 [M+Na]+.

*Carbons of the aromatic ring were not visible in the 13C NMR spectrum due to fluorine coupling.*

### Hydrolysis & Decarboxylation to Synthesise 5a-h,j-l,n,o

#### Hydrolysis & Decarboxylation General Method 1

The reduced Meldrum’s derivative (**4**) was dissolved in pyridine/water (5:2, 0.5 mmol/mL) and refluxed overnight. The reaction mixture was acidified with 1 M aqueous HCl (2 mL/mmol) and the resulting precipitate was filtered, washed with water and dried at the pump to afford the propionic acid derivatives without further purification.

#### Hydrolysis & Decarboxylation General Method 2

The reduced Meldrum’s derivative (**4**) was dissolved in pyridine/water (5:2, 0.5 mmol/mL), stirred at room temperature for 1 hr and refluxed for 4 hrs. The reaction mixture was acidified with 1M aqueous HCl (2 mL/mmol) and the resulting precipitate was filtered, washed with water and dried at the pump to afford the propionic acid derivatives without further purification.

#### 3-(4-Nitrophenyl)propanoic acid (5a)

Hydrolysis & Decarboxylation General Method 1. White solid (0.63 g, 65%); Mp 169-172 °C; 1H NMR (300 MHz, d6-DMSO) δ 12.23 (s, 1H, O*H*), 8.15 (d, *J* = 8.6 Hz, 2H, Ar), 7.53 (d, *J* = 8.6 Hz, 2H, Ar), 2.96 (t, *J* = 7.5 Hz, 2H, C*H*2), 2.62 (t, *J* = 7.5 Hz, 2H, C*H2*); 13C NMR (101 MHz, d6-DMSO) δ 173.9, 149.8, 146.4, 130.1, 123.9, 34.8, 30.5; IR (neat) ν (cm-1) 2915 (OH), 1696 (C=O); TOF MS EI+ m/z: 195.0 [M]+, 149.0 [C8H7NO2]+.

#### 3-(4-(Dimethylamino)phenyl)propanoic acid (5b)

Hydrolysis & Decarboxylation General Method 1. Orange solid (1.52 g, 91%); Mp 102-103 °C; 1H NMR (300 MHz, CDCl3) δ 7.09 (d, *J* = 8.7 Hz, 2H, Ar), 6.71 (d, *J* = 8.7 Hz, 2H, Ar), 2.93 – 2.83 (m, 8H, C*H3* & C*H2*), 2.68 – 2.59 (m, 2H, C­*H2*); 13C NMR (101 MHz, CDCl3) δ 179.1, 149.2, 129.1, 113.4, 113.0, 41.1, 36.1, 29.8; IR (neat) ν (cm-1) 2970 (OH), 1699 (C=O); TOF MS EI+ m/z: 193.1 [M+H]+, 134.1 [M-O2C2H3]+.

#### 3-(4-Methoxyphenyl)propanoic acid (5c)

Hydrolysis & Decarboxylation General Method 2. White solid (2.47 g, 93%); Mp 99-100 °C; 1H NMR (300 MHz, CDCl3) δ 7.13 (d, *J* = 8.6 Hz, 2H, Ar), 6.84 (d, *J* = 8.6 Hz, 2H, Ar), 3.79 (s, 3H, C*H3*), 2.90 (d, *J* = 7.9 Hz, 2H, C*H2*), 2.65 (d, *J* = 7.9 Hz, 2H, C*H2*); 13C NMR (101 MHz, CDCl3) δ 179.2, 158.1, 132.2, 129.2, 114.0, 55.3, 35.9, 29.7; IR (neat) ν (cm-1) 2930 (OH), 1699 (C=O); TOF MS ES- m/z: 179.1 [M-H]-; HR-MS calc. [C10H11O3]- 179.0714, obs. 179.0711.

#### 3-(Anthracen-9-yl)propanoic acid (5d)

Hydrolysis & Decarboxylation General Method 1. Orange solid (1.48 g, 80%*); Mp 193-195 °C; 1H NMR (300 MHz, d6-DMSO) δ 8.51 (s, 1H, Ar), 8.33 (dd, *J* = 8.5, 1.4, 2H, Ar), 8.10 (dd, *J* = 8.5, 1.4, 2H, Ar), 7.74 – 7.37 (m, 4H, Ar), 4.01 – 3.78 (m, 2H, ArC*H*2), 3.38 (s, 1H, O*H*), 2.78 – 2.58 (m, 2H, C*H2*COOH); 13C NMR (101 MHz, d6-DMSO) δ 174.2, 133.4, 131.6, 129.5, 129.5, 126.5, 126.4, 125.6, 124.5, 35.7, 23.2; IR (neat) ν (cm-1) 3050 (OH), 1688 (C=O); Scan ES- m/z: 249.1 [M-H]-.

**Yield over 2 steps*

#### 3-(Furan-2-yl)propanoic acid (5e)

Hydrolysis & Decarboxylation General Method 1. Black solid (0.58 g, ≤94%); 1H NMR (300 MHz, CDCl3) δ 10.91 (s, 1H, O*H*), 7.31 (dd, *J* = 1.9, 1.0 Hz, 1H, *H*C=CH), 6.28 (dd, *J* = 3.2, 1.9 Hz, 1H, *H*C=CH), 6.04 (dd, *J* = 3.2, 1.0 Hz, 1H, *H*C=CH), 2.97 (d, *J* = 7.6 Hz, 2H, C*H*2), 2.72 (t, *J* = 7.6 Hz, 2H, C*H*2); IR (neat) ν (cm-1) 3030 (OH), 1705 (C=O); TOF MS EI+ m/z: 140.1 [M]+.

#### 3-(Pyren-1-yl)propanoic acid (5f)

Hydrolysis & Decarboxylation General Method 1. Ochre solid (1.12 g, 98%); Mp 163-167 °C; 1H NMR (300 MHz, d3-MeOD) δ 8.30 (d, *J* = 9.5 Hz, 1H, Ar), 8.22 – 8.07 (m, 6H, Ar), 8.01 (d, *J* = 9.5 Hz, 1H, Ar), 7.92 (d, *J* = 7.9 Hz, 1H, Ar), 3.65 (t, *J* = 8.0 Hz, 2H, C*H2*), 2.83 (t, *J* = 8.0 Hz, 2H, C*H2*); 13C NMR (101 MHz, d3-MeOD) δ 175.2, 134.7, 131.4, 130.8, 130.1, 128.3, 127.2, 127.1, 126.7, 126.4, 125.6, 124.8, 124.6, 124.5, 124.5, 123.1, 122.6, 35.5, 28.2; IR (neat) ν (cm-1) 2903 (OH), 1690 (C=O); TOF MS ES- m/z: 273.3 [M-H]-.

#### 3-Cyclohexylpropanoic acid (5g)

Hydrolysis & Decarboxylation General Method 2. Colourless oil (88mg, 57%); 1H NMR (300 MHz, CDCl3) δ 11.51 (s, 1H, COO*H*), 2.36 (t, *J* = 7.8 Hz, 2H, CH2C*H2*COOH), 1.79-1.59 (m, 5H, Cy) 1.54 (q, *J* = 7.4 Hz, 2H, C*H2*CH2COOH), 1.32-1.06 (m, 4H, Cy), 0.98-0.84 (m, 2H, Cy); 13C NMR (101 MHz, CDCl3) δ 181.0, 37.1, 32.9, 32.0, 31.7, 26.5, 26.2; IR(neat) v (cm-1) 2923(OH), 1708 (C=O); TOF MS EI+ m/z: 157.0 [M+H]+.

#### 3-(p-Tolyl)propanoic acid (5h)

Hydrolysis & Decarboxylation General Method 2. White solid (1.54 g, 81%); Mp 116-118 °C; 1H NMR (300 MHz, CDCl3) δ 10.67 (s, 1H, O*H*), 7.10 (s, 4H, Ar), 2.92 (t, *J* = 7.8 Hz, 2H, C*H*2), 2.67 (d, *J* = 7.8 Hz, 2H, C*H2*), 2.32 (s, 3H, C*H*3); 13C NMR (101 MHz, CDCl3) δ 179.2, 137.1, 135.9, 129.2, 128.1, 35.7, 30.2, 21.0; IR (neat) ν (cm-1) 2919 (OH), 1698 (C=O); TOF MS ES- m/z: 163.0 [M-H]-; HR-MS calc. [C10H11O2]- 163.0765, obs. 163.0758.

#### 3-(2-Ethynylphenyl)propanoic acid (5j)

Hydrolysis & Decarboxylation General Method 1. White solid (0.14 g, ≤86%); 1H NMR (400 MHz, CDCl3) δ 7.49 (dd, *J* = 7.6, 1.4 Hz, 1H, Ar), 7.33 – 7.16 (m, 3H, Ar), 3.29 (s, 1H, C≡C*H*), 3.14 (d, *J* = 8.1 Hz, 2H, C*H2*), 2.75 (d, *J* = 8.1 Hz, 2H, C*H2*); IR (neat) ν (cm-1) 3287 (C≡C*H*), 2919 (OH), 1704 (C=O); TOF MS AP+ m/z: 175.1 [M+H]+.

#### 3-(2-bromophenyl)propanoic acid (5k)

Hydrolysis & Decarboxylation General Method 2. Yellow solid (65mg, 81%); Mp 97 °C; 1H NMR (300 MHz, CDCl3) δ 11.31 (s, 1H, COO*H*), 7.54 (d, *J* = 7.7 Hz, 1H, Ar), 7.35 – 7.16 (m, 2H, Ar), 7.09 (m, 1H, Ar), 3.08 (d, *J* = 7.1 Hz, 2H, C*H2*), 2.72 (dd, *J* =7.1 Hz, 2H, C*H2*); 13C NMR (101 MHz, CDCl3) δ 178.3, 139.4, 133.0, 130.4, 128.2, 127.6, 124.4, 33.8, 31.1; IR(neat) v (cm-1) 2905 (OH), 1697 (C=O); TOF MS EI+ m/z: 228.30/230.0 [M+H]+.

#### 3-(1H-Indol-3-yl)propanoic acid (5l)

Hydrolysis & Decarboxylation General Method 1. Red solid (66 mg, 48%); Mp 122-124 °C; 1H NMR (400 MHz, d3-MeOD) δ 7.52 (dt, *J* = 7.8, 1.0 Hz, 1H, Ar), 7.31 (dt, *J* = 8.1, 1.0 Hz, 1H, Ar), 7.10 – 6.96 (m, 3H, Ar), 3.05 (t, *J* = 7.4 Hz, 2H, C*H2*), 2.67 (t, *J* = 7.4 Hz, 2H, C*H2*); 13C NMR (101 MHz, d3-MeOD) δ 176.0, 136.7, 127.1, 121.4, 120.9, 118.1, 117.7, 113.6, 110.8, 34.7, 20.4; IR (neat) ν (cm-1) 3392 (NH), 3053 (OH), 1687 (C=O); TOF MS ES- m/z: 188.1 [M-H]-.

#### 3-(4-(Trifluoromethyl)phenyl)propanoic acid (5n)

Hydrolysis & Decarboxylation General Method 1. Viscous oil (0.34 g, 63%); 1H NMR (300 MHz, d3-MeOD) δ 7.59 (d, *J* = 8.0 Hz, 2H, Ar), 7.45 (d, *J* = 8.0 Hz, 2H, Ar), 3.02 (t, *J* = 7.6 Hz, 3H, ArC*H2*), 2.67 (t, *J* = 7.6 Hz, 2H. C*H2*COOH); 19F NMR (282 MHz, d3-MeOD) δ -63.90; 13C NMR (101 MHz, d3-MeOD) δ 174.8, 145.5, 129.3, 128.6, 124.9, 34.7, 30.3; IR (neat) ν (cm-1) 2901 (OH), 1707 (C=O); TOF MS EI+ m/z: 218.1 [M]+, 172.1 [M-CH2O2]+, 159.1 [M-C2H3O2]+.

*One carbon attributed to the CF3 was not visible in the 13C NMR spectrum.*

#### 3-(Perfluorophenyl)propanoic acid (5o)

Hydrolysis & Decarboxylation General Method 1. White solid (0.45 g, 91%); 1H NMR (300 MHz, d3-MeOD) δ 3.05 (t, *J* = 7.7 Hz, 2H, ArC*H2*), 2.63 (t, *J* = 7.7 Hz, 2H, C*H*2COOH); 19F NMR (282 MHz, d3-MeOD) δ -145.65 – -146.12 (m), -160.81 (t, *J* = 20.0 Hz), -165.89 – -166.27 (m); 13C NMR (101 MHz, d3-MeOD) δ 173.8, 144.1 (m), 138.9 (m), 137.3 (m), 114.1 (m), 32.4, 17.5; IR (neat) ν (cm-1) 2866 (OH), 1700 (C=O); TOF MS EI+ m/z: 240.1 [M]+, 194.1 [M-CHO2]+, 181.0 [M-C2H3O2]+.

## Carboxylic Acid Reduction to Synthesise 6a-h,j,l,n,o

### Carboxylic Acid Reduction General Method 1

Lithium aluminium hydride (1.2 equiv.) was added slowly to a solution of 3-propionic acid derivative (1.0 equiv.) in THF (0.2 M) at 0 °C, the reaction was allowed to reach room temperature and stirred for 16 hrs and quenched by the sequential addition of water (1 mL/mg LiAlH4), 2M aqueous NaOH (1 mL/mg LiAlH4) and a second portion of water (1 mL/mg LiAlH4) and allowed to stir for 30 mins. The reaction mixture was dried over MgSO4, filtered and evaporated under reduced pressure to afford the desired 3-propanol derivatives without further purification.

### Carboxylic Acid Reduction General Method 2

Borane in THF (7.7 mL, 7.7 mmol, 3.0 equiv.) was added dropwise to a solution of 3-propionic acid (1.0 equiv.) in THF (0.5 M) at 0 °C, warmed to room temperature and stirred for 4 hrs. The reaction mixture was quenched with 1 M aqueous HCl (20 mL/mmol), extracted with Et2O (2 x 100mL) and the combined organic phases were dried over MgSO4, filtered and the solvent was removed under reduced pressure. The residue was purified by column chromatography (silica, 0-100% EtOAc/hexane) to give the title compound.

#### 3-(4-Nitrophenyl)propan-1-ol (6a)

Carboxylic Acid Reduction General Method 2. Pale yellow oil (0.40 g, 86%); 1H NMR (400 MHz, CDCl3) δ 8.15 (d, *J* = 8.6 Hz, 2H, Ar), 7.37 (d, *J* = 8.6 Hz, 2H, Ar), 3.70 (t, *J* = 6.2 Hz, 2H, C*H2*), 2.84 (t, *J* = 7.8 Hz, 2H, C*H2*), 1.98 – 1.86 (m, 2H, C*H2*), 1.65 (s, 1H, OH); 13C NMR (101 MHz, CDCl3) δ 149.9, 146.4, 129.3, 123.7, 61.7, 33.6, 32.0; IR (neat) ν (cm-1) 3346 (OH); TOF MS EI+ m/z: 181.1 [M]+, 163.1 [M-H2O]+.

#### 3-(4-(Dimethylamino)phenyl)propan-1-ol (6b)

Carboxylic Acid Reduction General Method 1. Brown oil (1.30 g, 93%); 1H NMR (300 MHz, CDCl3) δ 7.08 (d, *J* = 8.6 Hz, 2H, Ar), 6.70 (d, *J* = 8.6 Hz, 2H, Ar), 3.67 (t, *J* = 6.4 Hz, 2H, C*H2*), 2.91 (s, 6H, N(C*­H3*)2), 2.61 (dd, *J* = 8.6, 6.4 Hz, 2H, C*H2*), 1.93 – 1.79 (m, 2H, C*H2*), 1.64 (s, 1H, O*H*); 13C NMR (101 MHz, CDCl3) δ 149.1, 129.9, 129.0, 113.1, 62.5, 40.9, 34.5, 31.0; IR (neat) ν (cm-1) 3310 (OH); TOF MS EI+ m/z: 179.2 [M]+, 134.1 [M- C2H5O]+.

#### 3-(4-Methoxyphenyl)propan-1-ol (6c)

Carboxylic Acid Reduction General Method 1. Yellow oil (1.80 g, 95%); 1H NMR (300 MHz, CDCl3) δ 7.11 (d, *J* = 8.7 Hz, 2H, Ar), 6.84 (d, *J* = 8.7 Hz, 2H, Ar), 3.78 (s, 3H, C*H3*), 3.65 (t, *J* = 6.4 Hz, 2H, C*H2*), 2.64 (d, *J* = 8.7 Hz, 2H, C*H2*), 1.95 – 1.70 (m, 3H, C*H2* & O*H*); 13C NMR (101 MHz, CDCl3) δ 157.8, 133.9, 129.3, 113.8, 62.2, 55.3, 34.4, 31.1; IR (neat) ν (cm-1) 3352 (OH); TOF MS EI+ m/z: 166.1 [M]+; HR-MS calc. [C10H14O2]+ 166.0988, obs. 166.0995.

#### 3-(Anthracen-9-yl)propan-1-ol (6d)

Carboxylic Acid Reduction General Method 1. Pale yellow solid (1.39 g, 99%); Mp 93-94 °C; 1H NMR (300 MHz, CDCl3) δ 8.44 – 8.20 (m, 3H, Ar), 8.11 – 7.89 (m, 2H, Ar), 7.66 – 7.35 (m, 4H, Ar), 3.82 (t, *J* = 6.2 Hz, 2H, C*H2*), 3.77 – 3.68 (m, 2H, C*H2*), 2.23 – 1.98 (m, 2H, C*H2*), 1.58 (s, 1H, O*H*); 13C NMR (101 MHz, CDCl3) δ 134.3, 131.6, 129.7, 129.2, 125.8, 125.6, 124.9, 124.3, 62.7, 34.0, 24.1; IR (neat) ν (cm-1) 3323 (OH); Scan ES+ m/z: 259.4 [M+H]+.

#### 3-(Pyren-1-yl)propan-1-ol (6f)

Carboxylic Acid Reduction General Method 1. Brown oil (1.03 g, 99%); 1H NMR (300 MHz, CDCl3) δ 8.15 (d, *J* = 9.3 Hz, 1H, Ar), 8.09 – 8.01 (m, 2H, Ar), 8.01 – 7.83 (m, 5H, Ar), 7.73 (d, *J* = 7.8 Hz, 1H, Ar), 3.64 (t, *J* = 6.3 Hz, 2H, C*H2*), 3.40 – 3.16 (m, 2H, C*H2*), 2.71 (s, 1H, O*H*), 1.99 (m, 2H, C*H*2); 13C NMR (101 MHz, CDCl3) δ 136.1, 131.4, 130.9, 129.9, 128.7, 127.8, 127.5, 127.3, 127.2, 126.7, 125.9, 125.3, 124.9, 124.9, 124.8, 123.3, 62.4, 34.5, 29.6; IR (neat) ν (cm-1) 3299 (OH); TOF MS ES+ m/z: 283.3 [M+Na]+.

#### 3-Cyclohexylpropan-1-ol (6g)

Carboxylic Acid Reduction General Method 1. Colourless oil (135mg, 57%); 1H NMR (300 MHz, CDCl3) δ 3.59 (t, *J* = 6.8 Hz, 2H, C*H2*), 2.48 (s, 1H, O*H*), 1.76 – 1.44 (m, 7H, Cy & C*H2*), 1.33 – 1.05 (m, 6H, Cy), 0.87 (m, 2H, Cy); 13C NMR (101 MHz, CDCl3) δ 63.19, 37.49, 33.42, 33.35, 33.21, 30.06, 26.66, 26.45, 26.36; IR(neat) v (cm-1) 3341(OH); TOF MS EI+ m/z: 142.1 [M]+.

#### 3-(p-Tolyl)propan-1-ol (6h)

Carboxylic Acid Reduction General Method 1. Yellow oil (1.23 g, 83%); 1H NMR (300 MHz, CDCl3) δ 7.07 (s, 4H, Ar), 3.62 (t, *J* = 6.5 Hz, 2H, C*H2*), 2.69 – 2.57 (m, 2H, C*H2*), 2.30 (s, 3H, C*H3*), 1.92 – 1.76 (m, 2H, C*H2*); 13C NMR (101 MHz, CDCl3) δ 138.8, 135.3, 129.1, 128.4, 62.1 , 34.4, 31.7, 21.0; IR (neat) ν (cm-1) 3324 (OH); TOF MS EI+ m/z: 150.1 [M]+, 132.1 [M-H2O]+; HR-MS calc. [C10H14O]+ 150.1039, obs. 150.1043.

#### 3-(2-Ethynylphenyl)propan-1-ol (6j)

Carboxylic Acid Reduction General Method 1. Colourless oil (63 mg, 53%); 1H NMR (400 MHz, CDCl3) δ 7.48 (dd, *J* = 7.6, 1.5 Hz, 1H, Ar), 7.30 – 7.20 (m, 2H, Ar), 7.16 (td, *J* = 7.6, 1.5 Hz, 1H, Ar), 3.66 (t, *J* = 6.4 Hz, 2H, C*H2*), 3.27 (s, 1H, C≡C*H*), 2.89 (t, *J* = 7.6 Hz, 2H, C*H2*), 1.97 – 1.86 (m, 2H, C*H2*), 1.78 (s, 1H, O*H*); 13C NMR (101 MHz, CDCl3) δ 144.4, 133.0, 129.0, 128.9, 125.9, 121.5, 82.4, 80.8, 62.1, 33.4, 30.5; IR (neat) ν (cm-1) 3291 (OH); TOF MS EI+ m/z: 160.1 [M]+, 141.1 [M-H2O]+.

#### 3-(2-Bromophenyl)propan-1-ol (6k)

Carboxylic Acid Reduction General Method 2. Colourless oil (92mg, 74%); 1H NMR (300 MHz, CDCl3) δ 7.54 (d, *J* = 7.7 Hz, 1H, Ar), 7.33 – 7.15 (m, 2H, Ar), 7.06 (m, 1H, Ar), 3.70 (t, *J* = 6.4 Hz, 2H, C*H2*), 2.84 (m, 2H, C*H2*), 2.04 – 1.76 (m, 2H, C*H2*); 13C NMR (75 MHz, CDCl3) δ 141.1, 132.8, 130.4, 127.7, 127.5, 124.5, 62.2, 32.8, 32.4; IR(neat) v (cm-1) 3340(OH); TOF MS EI+ m/z: 214/216 [M]+; HR-MS calc. [C9H11OBr79]+ 213.9988, obs. 213.9984.

#### 3-(1H-Indol-3-yl)propan-1-ol (6l)

Carboxylic Acid Reduction General Method 1. Orange oil (30 mg, 66%); 1H NMR (400 MHz, CDCl3) δ 8.01 (s, 1H, N*H*), 7.60 (dd, *J* = 7.8, 1.1 Hz, 1H, Ar), 7.33 (dt, *J* = 8.1, 1.1 Hz, 1H, Ar), 7.23 – 7.14 (m, 1H, Ar), 7.15 – 7.06 (m, 1H, Ar), 6.97 – 6.92 (m, 1H, Ar), 3.70 (t, *J* = 6.4 Hz, 2H, C*H2*), 2.84 (t, *J* = 7.0 Hz, 2H, C*H2*), 2.03 – 1.91 (m, 2H, C*H2*), 1.66 (s, 1H, O*H*); 13C NMR (101 MHz, CDCl3) δ 136.0, 127.5, 122.0, 121.6, 119.2, 118.9, 115.9, 111.2, 62.6, 32.9, 21.4; IR (neat) ν (cm-1) 3320 (OH); TOF MS AP+ m/z: 176.1 [M+H]+, 158.1 [M- H2O]+.

## Swern Oxidation to Synthesise 7a,c,d,f,g,h,j,k,o

DMSO (4.4 equiv.) in CH2Cl2 (0.2 M) was added dropwise to oxalyl chloride (2.2 equiv.) in CH2Cl2 (1 M) at -78 °C and stirred for 15 mins. Alcohol (1.0 equiv.) in CH2Cl2 (1 M) was added dropwise to the DMSO/oxalyl chloride solution and stirred for 1 hr at -78 °C. Triethylamine (5.0 equiv.) was added dropwise to the reaction mixture, stirred for 15 mins then slowly warmed to room temperature over 1 hr. Silica was added to the reaction mixture, the solvent evaporated under reduced pressure and the residue was purified by column chromatography (silica, 0-50% EtOAc/hexane) to give the aldehydes.

#### 3-(4-Nitrophenyl)propanal (7a)

White solid (307 mg, 84%); 1H NMR (400 MHz, CDCl3) δ 9.84 (s, 1H, C*H*O), 8.15 (d, *J* = 8.7 Hz, 2H, Ar), 7.37 (d, *J* = 8.7 Hz, 2H, Ar), 3.07 (t, *J* = 7.4 Hz, 2H, ArC*H2*), 2.87 (t, *J* = 7.4 Hz, 2H, C*H2*CHO); 13C NMR (101 MHz, CDCl3) δ 200.1, 148.3, 146.6, 129.3, 123.8, 44.5, 27.8; IR (neat) ν (cm-1) 1713 (C=O); TOF MS EI+ m/z: 179.0 [M]+.

#### 3-(4-Methoxyphenyl)propanal (7c)

Yellow oil (1.33 g, 84%); 1H NMR (300 MHz, CDCl3) δ 9.81 (t, *J* = 1.5 Hz, 1H, C*H*O), 7.11 (d, *J* = 8.8 Hz, 2H, Ar), 6.83 (d, *J* = 8.8 Hz, 2H, Ar), 3.78 (s, 3H, C*H*3), 2.97 – 2.85 (m, 2H, C*H*2), 2.87 – 2.65 (m, 2H, C*H*2); 13C NMR (101 MHz, CDCl3) δ 201.8, 158.1, 132.3, 129.2, 114.0, 55.3, 45.6, 27.3; IR (neat) ν (cm-1) 1721 (C=O); TOF MS EI+ m/z: 164.1 [M]+; HR-MS calc. [C10H12O2]+ 164.0832, obs. 164.0836.

#### 3-(Anthracen-9-yl)propanal (7d)

Yellow solid (2.34 g, 89%); Mp 84-86 °C; 1H NMR (300 MHz, CDCl3) δ 9.87 (s, 1H, C*H*O), 8.31 (s, 1H, Ar), 8.17 – 8.08 (m, 2H, Ar), 8.00 – 7.92 (m, 2H, Ar), 7.56 – 7.35 (m, 4H, Ar), 3.95 – 3.82 (m, 2H, ArC*H2*), 2.93 – 2.80 (m, 2H, C*H2*CHO); 13C NMR (101 MHz, CDCl3) δ 201.4, 134.0, 132.4, 131.6, 129.5, 126.4, 126.0, 125.0, 123.8, 44.9, 20.0; IR (neat) ν (cm-1) 1730 (C=O); TOF MS EI+ m/z: 234.1 [M]+, 191.1 [M-CH2CHO]+.

#### 3-(Pyren-1-yl)propanal (7f)

Yellow oil (0.60 g, 66%); 1H NMR (300 MHz, CDCl3) δ 9.84 (t, *J* = 1.3 Hz, 1H, C*H*O), 8.16 – 8.10 (m, 3H, Ar), 8.08 – 8.02 (m, 2H, Ar), 8.00 – 7.92 (m, 3H, Ar), 7.79 (d, *J* = 7.8 Hz, 1H, Ar), 3.59 (t, *J* = 7.7 Hz, 2H, ArC*H2*), 2.92 (td, *J* = 7.7, 1.3 Hz, 2H, C*H2*CHO); 13C NMR (101 MHz, CDCl3) δ 201.4, 134.3, 131.4, 130.8, 130.2, 128.5, 127.7, 127.5, 127.0, 126.9, 126.0, 125.2, 125.2, 125.0, 124.5, 123.5, 122.7, 45.5, 25.7; IR (neat) ν (cm-1) 1621 (C=O); TOF MS AP+ m/z: 258.1 [M]+, 241.1 [M-OH]+; HR-MS calc. [C19H14O]+ 258.1039, obs. 258.1050.

#### 3-Cyclohexylpropanal (7g)

Colourless oil (46mg, 46%); 1H NMR (300 MHz, CDCl3) δ 9.77 (t, *J* = 1.9 Hz, 1H, C*H*O), 2.43 (dt, *J* = 7.1, 1.9 Hz, 2H, C*H2*), 1.76 – 1.58 (m, 5H, Cy & C*H2*), 1.53 (dt, *J* = 7.1, 1.9 Hz, 2H, C*H2*), 1.29 – 1.13 (m, 4H, Cy), 0.97 – 0.82 (m, 2H, Cy); 13C NMR (101 MHz, CDCl3) δ 203.2, 114.3, 102.1, 41.5, 37.20, 33.0, 29.4, 26.5, 26.2; IR(neat) v (cm-1) 1737 (C=O); TOF MS EI+ m/z: 140 [M]+; HR-MS calc. [C9H16O]­+ 140.1196, obs. 140.1203.

#### 3-(p-Tolyl)propanal (7h)

Colourless oil (0.92 g, 83%); 1H NMR (300 MHz, CDCl3) δ 9.81 (t, *J* = 1.5 Hz, 1H, C*H*O), 7.09 (t, *J* = 2.3 Hz, 4H, Ar), 3.01 – 2.86 (m, 2H, C*H*2), 2.81 – 2.69 (m, 2H, C*H2*), 2.31 (s, 3H, C*H3*); 13C NMR (101 MHz, CDCl3) δ 201.7, 137.2, 135.8, 129.3, 128.2, 45.4, 27.7, 21.0; IR (neat) ν (cm-1) 1723 (C=O); TOF MS E+ m/z: 148.1 [M]+; HR-MS calc. [C10H12O]+ 148.0883, obs. 148.0886.

#### 3-(2-Ethynylphenyl)propanal (7j)

Colourless oil (46 mg, 77%); 1H NMR (400 MHz, CDCl3) δ 9.83 (t, *J* = 1.4 Hz, 1H, C*H*O), 7.49 (dd, *J* = 7.6, 1.4 Hz, 1H, Ar), 7.29 (td, *J* = 7.6, 1.4 Hz, 1H, Ar), 7.26 – 7.14 (m, 2H, Ar), 3.29 (s, 1H, C≡C*H*), 3.13 (t, *J* = 7.7 Hz, 2H, ArC*H2*), 2.82 (td, *J* = 7.7, 1.4 Hz, 2H, C*H2*CHO); 13C NMR (101 MHz, CDCl3) δ 201.5, 143.0, 133.1, 129.2, 128.9, 126.4, 121.5, 81.9, 81.4, 44.4, 27.0; IR (neat) ν (cm-1) 3285 (CCH), 1718 (C=O); TOF MS EI+ m/z: 158.1 [M]+, 130.1 [M-H2O]+.

#### 3-(2-bromophenyl)propanal (7k)

Colourless oil (67mg, 75%); 1H NMR (400 MHz, CDCl3) δ 9.83 (t, *J* = 1.3 Hz, 1H, C*H*O), 7.71 – 7.42 (m, 1H, Ar), 7.28 – 7.17 (m, 2H, Ar), 7.08 (m, 1H, Ar), 3.06 (t, *J* = 7.6 Hz, 2H, C*H2*), 2.80 (t, *J* = 7.5, 2H, C*H2*); 13C NMR (101 MHz, CDCl3) δ 201.0, 139.7, 133.0, 130.5, 128.1, 127.7, 124.3, 43.7, 28.7; IR(neat) v (cm-1) 1720 (C=O), 1471 (C=C); TOF MS EI+ m/z: 212/214 [M]+; HR-MS calc. [C9H9OBr79]+ 211.9831, obs. 211.9841.

#### 3-(Perfluorophenyl)propanal (7o)

Colourless oil (94 mg, 29%); 1H NMR (400 MHz, CDCl3) δ 9.81 (s, 1H, C*H*O), 3.03 (t, *J* = 7.5 Hz, 1H), 2.80 (t, *J* = 7.5 Hz, 1H); 19F NMR (282 MHz, CDCl3) δ -143.55 – -143.70 (m), -156.88 (t, *J* = 20.8 Hz), -162.26 – -162.55 (m); 13C NMR (101 MHz, CDCl3) δ 199.5, 143.8 (m), 139.1 (m), 136.3 (m), 113.4 (td, *J* = 18.8, 3.4 Hz), 42.5, 15.1; IR(neat) v (cm-1) 1729 (C=O), 1499 (C=C); TOF MS EI+ m/z: 224.1 [M]+.
